# Gene silencing of indoleamine 2,3-dioxygenase 2 in melanoma cells induces apoptosis through the suppression of NAD+ and inhibits *in vivo* tumor growth

**DOI:** 10.18632/oncotarget.8617

**Published:** 2016-04-06

**Authors:** Yanling Liu, Yujuan Zhang, Xiufen Zheng, Xusheng Zhang, Hongmei Wang, Qin Li, Keng Yuan, Nanjing Zhou, Yanrong Yu, Na Song, Jiamin Fu, Weiping Min

**Affiliations:** ^1^ Institute of Immunotherapy of Nanchang University, and Jiangxi Academy of Medical Sciences, Nanchang, China; ^2^ Jiangxi University of Technology, Nanchang, China; ^3^ Jiangxi Provincial Key Laboratory of Immunotherapy, Nanchang, China; ^4^ Department of Surgery, Pathology, and Oncology, University of Western Ontario, London, Canada

**Keywords:** IDO2, NAD+, apoptosis, neoplasm, siRNA

## Abstract

Indoleamine 2,3-dioxygenase 2 (IDO2) is a newly discovered enzyme that catalyzes the initial and rate-limiting step in the degradation of tryptophan. As a homologous protein of IDO1, IDO2 plays an inhibitory role in T cell proliferation, and it is essential for regulatory T cell (Treg) generation in healthy conditions. Little is known about the immune-independent functions of IDO2 relevant to its specific contributions to physiology and pathophysiology in cancer cells. The purpose of this study was to assess the impact of IDO2 gene silencing as a way to inhibit B16-BL6 cancer cells in a murine model. Here, for the first time, we show that knockdown of IDO2 using small interfering RNA (siRNA) inhibits cancer cell proliferation, arrests cell cycle in G1, induces greater cell apoptosis, and reduces cell migration *in vitro*. Knockdown of IDO2 decreased the generation of nicotinamide adenine dinucleotide (NAD+) while increasing the generation of reactive oxygen species (ROS). We further demonstrate that cell apoptosis, induced by IDO2 downregulation, can be weakened by addition of exogenous NAD+, suggesting a novel mechanism by which IDO2 promotes tumor growth through its metabolite product NAD+. In addition to *in vitro* findings, we also demonstrate that IDO2 silencing in tumor cells using short hairpin RNA (shRNA) delayed tumor formation and arrested tumor growth *in vivo*. In conclusion, this study demonstrates a new non-immune-associated mechanism of IDO2 *in vitro* and IDO2 expression in B16-BL6 cells contributes to cancer development and progression. Our research provides evidence of a novel target for gene silencing that has the potential to enhance cancer therapy.

## INTRODUCTION

Indoleamine 2,3-dioxygenase 2 (IDO2) is a newly discovered enzyme that can catabolize tryptophan into kynurenine [1–3]. IDO2, an isoform of IDO1, is located immediately downstream of classic IDO1 on human chromosome 8p21. IDO1 and IDO2 have similar genomic structure and function. Both of them are highly expressed in various types of cancer cells as well as immune cells, such as dendritic cells (DC) [2, 4]. Both IDO1 and IDO2 are known immunosuppressive molecules, and their immune suppressive function has been investigated both *in vivo* and *in vitro*. A recent study reported by the Koropatnick group shows that IDO1, expressed in tumor cells, presents immune-independent function in response to chemotherapeutic and radioactive drugs [5]. We recently observed that cells in the invasive melanoma cell line, B16-BL6, highly express IDO2 whereas the less invasive melanoma cell line, B16F10, had limited expression of IDO2 in the absence of interferon-gamma (IFN-γ) induction. Although this suggests that IDO2 may also possess non-immune function on tumor biological characters, the physiological and pathological roles of IDO2 remain unclear. The non-immune function of IDO2 has not been well investigated.

Previous studies have shown that 1-methyl-L-tryptophan (L-1-MT) is a more potent inhibitor of IDO1, while D stereoisomer (D-1-MT, selective for IDO2 [2]) is more effective inhibiting IDO1-expressing tolerogenic DC. D-1-MT was shown to have superior anti-tumor activity in preclinical models [6], suggesting the important role of IDO2 in cancer development. Controversially, it has also been reported that L-1-MT, rather than D-1-MT, inhibited IDO2 activity [7–9]. It is important to identify IDO2 selective inhibitors to understand IDO2 function. With the significant structural similarity shared by IDO1 and IDO2 and the same substrate utilized by them, it is difficult to develop distinct inhibitors. RNA interference (RNAi) using short interfering RNA (siRNA) offers an opportunity to exclusively study the role of IDO2 *in vitro* as siRNA can induce sequence-specific gene inhibition at the post-transcription level. Short hair RNA (shRNA) provides the opportunity to study IDO2 *in vivo*.

Tryptophan, an essential amino acid, can be degraded to a series of physiological materials that are required in several physiological processes. Both IDO1 and IDO2 have the enzymatic ability to catabolize tryptophan into kynurenine, which is a supplier of NAD+. As an essential co-factor required in many biochemical processes, NAD+ is consumed by tumor cells at higher rates. Inhibition of NAD+ formation leads to cell apoptosis [10, 11]. Knocking down IDO1 using siRNA-reduced NAD+ generation, leads to enhanced sensitivity of tumor cells to chemotherapeutic drugs [5]. It is unknown, however, if downregulation of IDO2 expression can induce cell apoptosis/death by suppressing NAD+. Moreover, the potential to knockdown IDO2 using RNAi to prevent tumor growth has not been explored.

In this study, we investigate the biological function of IDO2 in melanoma cancer cells using RNAi to elucidate the non-immune function of IDO2 and its mechanism in tumor growth. We also evaluate the potential therapeutic effect of IDO2 siRNA and shRNA in a murine melanoma model.

## RESULTS

### IDO2 siRNA significantly knocks down IDO2 expression in B16-BL6 cells, but does not affect IDO1 expression

**Figure 1:**
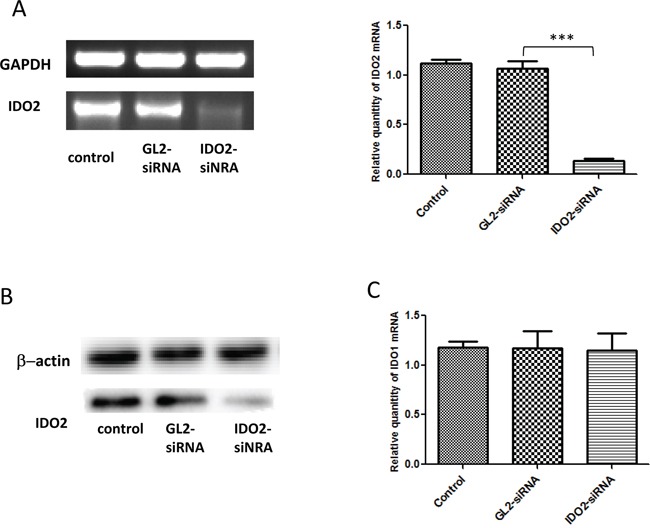
Gene silencing of IDO2 with siRNA in B16-BL6 cells **A.** Silencing IDO2 in B16-BL6 cell line: B16-BL6 cells were transfected with different siRNA targeting IDO2 or GL2 (control siRNA), or remained untransfected as a blank control. IDO2 mRNA expression levels were measured 24 h post transfection by conventional RT-PCR (left panel) and qRT-PCR (right panel) as described in Materials and Methods. Bars indicate the means of two independent measurements (n=3 for each measurement) ± SD (***p≤0.001). **B.** IDO2 expression at the protein level: B16-BL6 cells were transfected with IDO2 or GL2 siRNA, or remained untransfected as a blank control. After 48 h transfection, total protein was extracted from cells and separated by PAGE. IDO2 protein expression level in B16-BL6 cells was detected by Western blot. **C.** IDO2 siRNA did not affect IDO1 expression. IDO1 mRNA expression levels in the cells from (A) were measured 24 h post transfection by qPCR. Bars indicate the mean of three independent measurements ± SD.

Human primary tumors, such as gastric, colon and renal cell carcinomas, constitutively express IDO2 mRNA, whereas its expression in cancer cell lines has to be induced by IFN-γ [8]. Therefore, we assessed if IDO2 is expressed in mouse cancer cell lines. Initially, we measured IDO2 expression in a panel of mouse tumor cell lines of differing histological origin (data not shown). The murine melanoma cell line B16-BL6 showed strong expression of IDO2 without IFN-γ. To test the efficacy of gene silencing using IDO2 siRNA, B16-BL6 was transfected with IDO2 siRNA or GL2 siRNA (control siRNA). Twenty-four hours after transfection, IDO2 expression was measured at the transcriptional level by RT-PCR (Figure 1A, left panel). In the cells with IDO2 siRNA transfection, IDO2 mRNA expression decreased by >80% (Figure 1A, right panel). IDO2 expression at the protein level was also measured by Western blot analysis (Figure 1B). Data showed that the designed IDO2 siRNA effectively downregulated expression of IDO2 in B16-BL6 cells.

Because IDO1 gene is located upstream of IDO2 gene on chromosome 8 and these two genes possess adjacent structural and evolutionary relationships, genetic knockdown one of them may cause a change in expression of the other [1, 2]. Moreover, alternative splice variants of IDO2 mRNA are found in macrophages which obtained from IDO1 ko mice [12]. To investigate if IDO2 gene silencing changes IDO1 expression, we measured the expression of IDO1 by qPCR. Data showed that IDO2 siRNA did not affect expression of IDO1 (Figure 1C).

### Biological changes occur in B16-BL6 cells after IDO2 gene silencing

**Figure 2:**
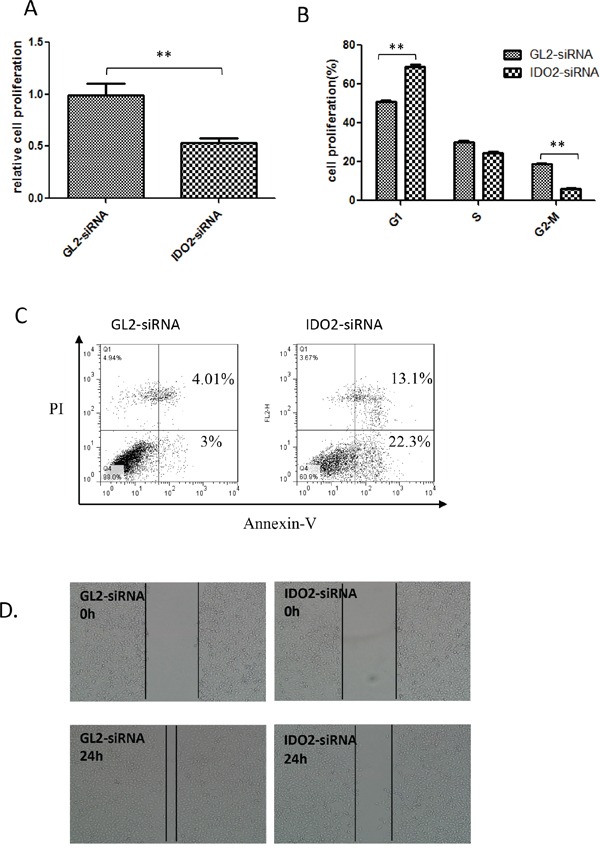
Impact of IDO2 on the biological activities of B16-BL6 cells **A.** IDO2 gene silencing slowed the proliferation of B16-BL6 cells. After IDO2 or GL2 siRNA transfection, B16-BL6 cell proliferation was measured by MTT. Bars indicate the mean of five measurements ± SD (**p≤0.01). **B.** Gene silencing of IDO2 arrests the cell cycle in G1: B16-BL6 cells were transfected with IDO2 or GL2 siRNA for 24 h. Cells were harvested and fixed overnight, followed by flow cytometry analyses of cells after staining with PI. **C.** IDO2 silencing increased cell apoptosis. B16-BL6 cells were transfected with IDO2 or GL2 siRNA. 48 h after transfection, cells were double stained with FITC-conjugated annexin V and PI and followed by flow cytometric analysis. **D.** IDO2 gene silencing inhibited the migration of B16-BL6 cells. B16-BL6 cells were transfected with IDO2 or GL2 siRNA for 4 h and then were scratched. Images of cell migration were taken at the beginning of observation and 24 h afterwards.

IDO1 expression correlates with decreased proliferation of immune cells [13, 14] and its silencing provided the opposite outcome [5]. Because IDO2 shares the same enzymatic function, we investigated the effect of IDO2 on B16-BL6 cell proliferation using an MTT assay. Silencing IDO2 significantly reduced proliferation of B16-BL6 cells (Figure 2A).

It is also reported that IDO1-mediated depletion of tryptophan induces cell cycle arrest in T cells at G1 [15]. We accordingly measured the effect of IDO2 siRNA on cell cycle. Interestingly, IDO2 gene silencing induced more cells in G1 in contrast to IDO1 (Figure 2B).

Tryptophan is an essential amino acid, and its depletion results in cell apoptosis [16, 17]. Although the enzymatic activity of IDO2 is not as great as IDO1, studies have demonstrated that both IDOs can catabolize tryptophan. Therefore, we used flow cytometry to detect if silencing IDO2 would reduce cell apoptosis. Annexin-V and propidium iodide (PI) double positive cells increased in IDO2 siRNA-transfected cells as compared to cells transfected with the control GL2 siRNA (Figure 2C).

B16-F10 and B16-BL6 are two variant cell lines of B16 melanoma with markedly different spontaneous metastatic behavior [18]. B16-BL6 is more invasive than B16-F10 and the two cell lines express differential levels of IDO2. We investigated if IDO2 expression contributes to differences in cell invasion by measuring cell migration distance using a cell scratch assay. As shown in Figure 2D, the gap created by scratch was almost filled with GL2 siRNA-transfected cells, whereas the gap still remained in IDO2 siRNA-transfected cells. The data indicate that silencing IDO2 can significantly inhibit tumor cell migration (Figure 2D).

### Gene silencing of IDO2 induces apoptosis and is associated with suppression of NAD+ generation and upregulation of ROS

**Figure 3:**
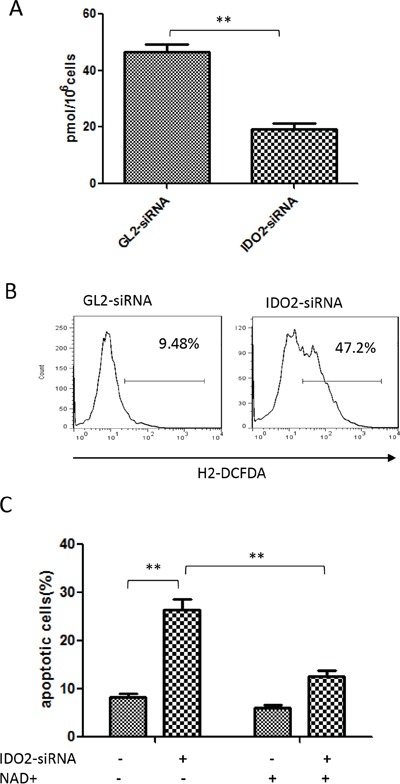
Gene silencing of IDO2–induced apoptosis is associated with suppression of NAD+ generation and upregulation of ROS in B16-BL6 cells **A.** Gene silencing of IDO2 decreased intracellular NAD+. B16-BL6 cells were transfected with IDO2 or GL2 siRNA for 48 h, or remained untransfected as blank control. NAD+ levels were measured as described in Materials and Methods. Bars indicate the mean of three independent measurements ± SD (**p≤0.01). **B.** Intracellular ROS increased in IDO2-silenced cells. B16-BL6 cells were transfected with IDO2 or GL2 siRNA for 48 h. ROS levels in different groups were measured by dichlorofluorescein assay and detected by flow cytometry. **C.** Exogenous NAD+ can relieve the apoptosis induced by IDO2 gene silencing. B16-BL6 cells were transfected with IDO2 or GL2 siRNA for 4 h, then treated with 100 μM NAD+. Cell apoptosis was measured 48 h after treatment with NAD+, as described in Figure 2A. Bars indicate the mean of three independent measurements ± SD (**p≤0.01).

IDO catalyzes the initial, rate-limiting step in tryptophan catabolism into the kynurenine pathway. In this step, L-tryptophan is converted to L-kynurenine, a source of NAD+ in cells. NAD+, in turn, can inhibit ROS generation by ketoglutarate dehydrogenase and pyruvate dehydrogenase [19]. In fact, tumor cells consume large amounts of NAD+ [10, 11]. We measured the intracellular NAD+ concentration and ROS generation after IDO2 gene silencing. We found that intracellular NAD+ concentration in the IDO2 gene-silenced cells was significantly lower than the GL2 control siRNA-transfected group (Figure 3A). We also found that IDO2 gene silencing significantly increased the ROS level in B16-BL6 cells (Figure 3B).

To understand if cell apoptosis, caused by IDO2 gene silencing, is attributed to the decrease of NAD+, exogenous NAD+ was supplemented to the B16-BL6 cells after IDO2 or GL2 siRNA transfection. As shown in Figure 3C, exogenous NAD+ rescued cells from apoptosis in IDO2-silenced B16-BL6 cells, suggesting NAD+ is involved in cell apoptosis induced by knockdown of IDO2.

### Knockdown of IDO2 in tumor cells reduces tumor growth and tumor formation *in vivo*

To determine the role of IDO2 in tumor growth *in vivo*, we first generated an IDO2 shRNA transfected stable cell line. IDO2 siRNA was cloned into a shRNA expressing vector containing a GFP reporter gene. We detected the fluorescence of GFP in the generated stable cell line by flow cytometry to confirm the purity of stable cells. As shown in Supplementary Figure 1, both IDO2 shRNA and scrambled shRNA transfected cells are GFP positive. Moreover, we measured the IDO2 expression in these stable cells by qPCR. The results show that there was 82% downregulation of IDO2 expression in IDO2 shRNA stable cells, but no reduction of IDO2 expression in the scrambled shRNA cells in comparison to B16-BL6 untransfected control cells (Supplementary Figure 2). IFN-γ is a known stimulating factor of IDO2 [8]. We treated IDO2 shRNA and scrambled shRNA cells or wild type B16-BL6 cells with IFN-γ and measured IDO2 expression. The results show that IDO2 expression was increased (6-fold change) in control and scrambled shRNA B16-BL6 cells stimulated with IFN-γ. However, IDO2 expression, stimulated by IFN-γ, was abrogated in the IDO2 shRNA transfected cells (Supplementary Figure 3).

**Figure 4:**
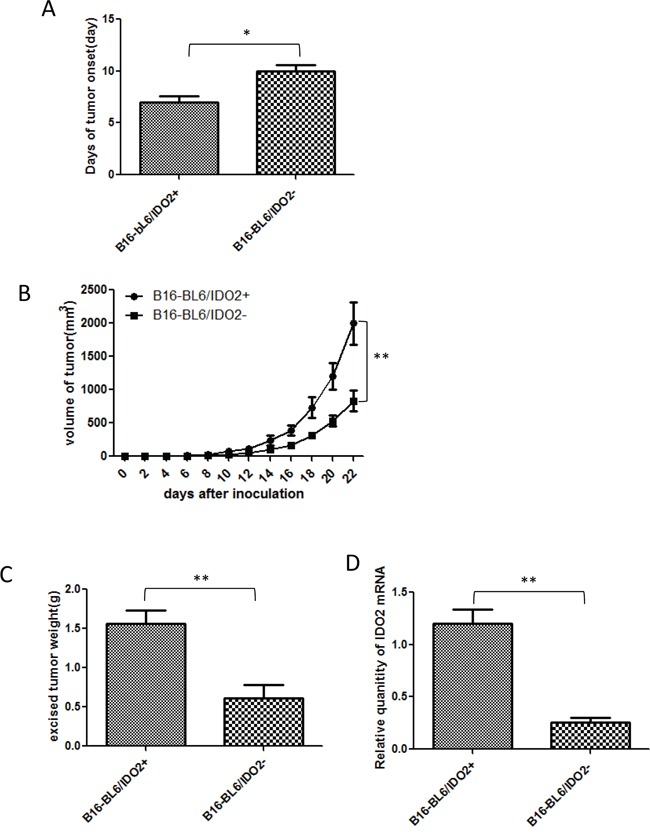
Impact of IDO2 in tumor formation and growth *in vivo* **A.** Knockdown of IDO2 in B16-BL6 cells delayed tumor onset. IDO2 shRNA or scrambled shRNA transfected stable B16-BL6 cells (2×10^5^cells), which were subcutaneously injected into the upper hind leg of C57BL/6 mice (n=12/group). Tumor onset day was defined as the time when tumor diameter reached 5 mm (*p≤0.05). **B.** Silencing IDO2 reduced tumor growth (**p≤0.01). **C.** Excised tumor weight of B16-BL6/IDO2- is lighter than B16-BL6/IDO2+. Tumors were excised at the end point of the experiment and weighed (**p≤0.01). **D.** IDO2 expression in tumor tissue. Total RNA was extracted from tumor tissue and IDO2 expression was measured by qRT-PCR.

Next we investigated the effect of differential expression levels of IDO2 in tumor cells on tumor formation and growth *in vivo*. We observed that tumor onset in IDO2 shRNA stable cells (B16-BL6/IDO2-) injected mice was substantially postponed compared with mice injected with scrambled shRNA cells (B16-BL6/IDO2+) (Figure 4A). In addition to tumor onset time, we found that tumor growth was constrained in IDO2 shRNA stable cells injected mice compared with scrambled shRNA stable cells injected mice (Figure 4B). At the end point of the observation, tumor tissues were excised and weighed. The mass of tumors derived from IDO2 shRNA stable cells was much smaller than those from the scrambled shRNA stable cell injected mice (Figure 4C). Furthermore, we found that IDO2 expression in IDO2 shRNA tumor tissue still maintained 80% gene silencing 22 days after cell inoculation (Figure 4D).

### Treatment of IDO2 shRNA arrests the development and progression of tumor *in vivo*

**Figure 5:**
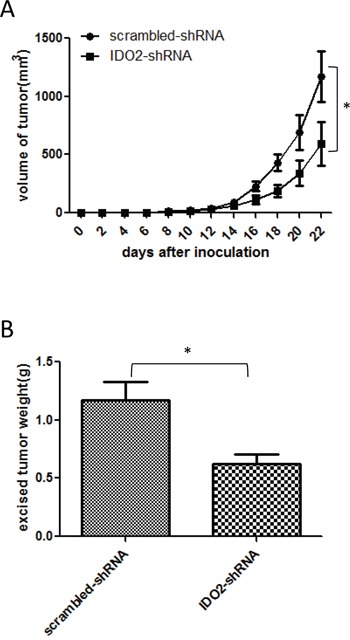
Treatment with IDO2 shRNA *in vivo* suppresses tumor growth C57BL/6 mice were treated with 50 μg of IDO2 shRNA or scrambled shRNA in 1 ml PBS by hydrodynamic injection through the tail vein three days before cancer cell inoculation when 2×10^5^ B16-BL6 cells were subcutaneously injected into the upper hind leg. At 7, 14 and 21 days after cancer cell inoculation, mice were treated with 50 μg IDO2 shRNA or scrambled vectors as described above. Groups of mice treated with scrambled shRNA were set as controls (*p<0.05 vs control groups). The tumor growth curve **A.** and the tumor weight **B.** were determined as described in Figure 4 (n=12; *p<0.05 vs control groups).

To explore the therapeutic effect of silencing IDO2, we treated tumor-bearing mice with IDO2 shRNA. As shown in Figure 5A, tumor growth was significantly slower in IDO2 shRNA treated mice compared with scrambled shRNA treated mice. Tumor weight derived from IDO2 shRNA treated mice was less than scrambled shRNA treated mice (Figure 5B). These results indicate that IDO2 silencing can inhibit tumor growth in a murine melanoma model.

## DISCUSSION

In this study, we demonstrated that siRNA knockdown of IDO2 inhibited cancer cell proliferation, arrested cell cycle in G1, induced greater apoptosis, and reduced cell migration *in vitro*. Knockdown of IDO2 also decreased the generation of kynurenine and NAD+ while increasing the generation of ROS. We further demonstrated that cell apoptosis, induced by IDO2 downregulation, can be attenuated by addition of exogenous NAD+, suggesting a novel mechanism by which IDO2 promotes tumor growth through its metabolite product NAD+. In addition to these *in vitro* findings, we also showed that using shRNA to silence IDO2 in tumor cells delayed tumor formation and arrested tumor growth *in vivo*, providing a potentially new therapy for cancer.

Both IDO1 and IDO2 are rate-limiting enzymes in the catabolization of tryptophan to kynurenine. It is well appreciated that IDO1 presents very high enzymatic activity, while it is uncertain about the extent of IDO2 activity. Most studies have shown it is difficult to measure IDO2 activity. In our study, B16-BL6 cells highly expressed IDO2, and IDO2 had sufficient enzymatic activity as shown by a reduction of kynurenine and it metabolite NAD+ in this pathway. The activity of IDO2 has been measured in stable transfected T-REK cells [20] and human DC [21]. The expression level or stability of IDO2 and cell culture conditions may affect its enzymatic activity. However, its specific contributions to normal physiology and pathophysiology are unknown. There are almost no reports of IDO2 function in tumor cells or tumor formation and growth. The data we show here reveals the new immune-independent function of IDO2 in mediating tumor formation and growth.

We investigated the effect of IDO2 on the physiological and pathophysiological functions of tumor cells, including cell proliferation, migration, apoptosis and cell cycle. Interestingly, IDO2 gene silencing slowed B16-BL6 cell proliferation, increased accumulation of cells in G1 and decreased accumulation in G2/M, which is in contrast to IDO1. It has been reported that IDO1 impedes tumor cell proliferation [22] and IDO1-mediated depletion of tryptophan induces cell cycle arrest in T cells at G1 [15]. This difference might be attributed to the reduction of NAD+, which resulted from silencing IDO2, as NAD+ is required and highly consumed by tumor cells. These data demonstrate that IDO2 plays an important role in cell biological progress and is not reduntant. Further study is needed to elucidate this phenomenon.

IDOs can degrade tryptophan to generate kynurenine, which then undergoes several degradation steps to produce quinolinic acid, a source of NAD+ in cells. Antisense-mediated reduction of IDO1 expression reduced intracellular NAD+ in A549 human tumor cell line by approximately 60% [23]. FK866, a pharmacological inhibitor of NAD+, has been tested in a phase II clinical trial for treatment of chronic B-cell lymphocytic leukemia and cutaneous T-cell lymphoma [24]. There was a similar reduction after treatment *in vitro* with cancer cells becoming sensitive to apoptosis after depletion of NAD+ [10]. Addition of exogenous NAD+ shows significant cytoprotection from apoptosis triggered by staurosporine, C2-ceramide, or N-methyl-N-nitro-N-nitrosoguanidine [11]. In addition, NAD+ can inhibit ROS generation from ketoglutarate dehydrogenase and pyruvatedehydrogenase [19]. Both NAD+ decrease and ROS increase can lead to cancer cell apoptosis [11]. In our study, IDO2 silencing resulted in a similar reduction in the amount of intracellular NAD+ in B16-BL6 cells with significantly elevated ROS levels. Our data further showed that addition of exogenous NAD+ rescued IDO2-silenced cells from apoptosis. Taken together, our data suggest that IDO2 knockdown-associated apoptotic signaling might be mediated by NAD+.

Like IDO1, IDO2 is an immunosuppressive molecule, and it plays an important role in induction and maintenance of tumor microenvironment immune tolerance [25–27]. IDO2 gene-transfected 293 cell line inhibited CD4+ and CD8+ T cell proliferation in a co-cultured system [28]. IDO2 not only directly enhances Treg generation, but it also facilitates the immunosuppressive function of IDO1. Systemic treatment with IDO2 shRNA may not only impair tumor function but it may also change the host immune system, leading to an overall reduction of tumor burden. The effect of IDO2 shRNA treatment will be a future research direction.

In summary, for the first time, this study demonstrates direct evidence of physiological and pathophysiological effects of IDO2 on B16 melanoma. Using siRNA to knockdown IDO2 expression in B16-BL6 cells, we have shown that the role of IDO2 in tumor development and progression is correlated with the production of NAD+ and ROS. IDO represents an ideal target for immunomodulation, and we have confirmed that IDO2 could be a new therapeutic target. IDO2 suppression through gene silencing is a promising strategy for effective cancer therapy.

## MATERIALS AND METHODS

### Animal and cell lines

Male C57BL/6 mice were purchased from The Jackson Laboratory. A murine melanoma cell line established from a C57BL/6 mouse and designated B16-BL6 was obtained from the American Type Culture Collection(ATCC)) and maintained in DMEM medium (Life Technologies, Carlsbad, CA) with 10% FBS, L-glutamine, penicillin, and streptomycin at 37°C in 5% CO_2_.

### siRNA synthesis and transfection

The siRNA targeting IDO2 mRNA was generated in accordance with the target sequence selection method described by Elbashir et al [29]. siRNA was synthesized by the manufacturer (Sigma, St. Louis, MO). SiRNA targeting luciferase gene GL2 (GL2 siRNA) was used as a scrambled-silencing control since GL2 is not expressed in treated cells. IDO2 siRNA and GL2 siRNA were transfected into B16-BL6 cells using lipofectamine 2000 reagent (Invitrogen, Burlington, ON, Canada) as described previously [30]. Briefly, cells were plated into 12-well plates (1.2 ×10^5^ cells/well) and allowed to grow overnight to reach 50-70% confluence. Cell medium was replaced with the 300 μl OptiMEM^®^ serum-reduced medium (Invitrogen Life Technologies, Carlsbad, CA) before transfection. 1 μg of IDO2 siRNA or GL2 siRNA was incubated with 2 μl of lipofectamine 2000 reagent in 200 μl of Optimal serum-reduced medium at room temperature for 20 min, and then the mixture was gently added to each group.

### Stable cell line construction

The siRNA expression vector (shRNA) for stable transfection was constructed as previously described [31, 32]. The oligonucleotides containing target-specific sense and anti-sense sequences of IDO2 mRNA were synthesized, annealed and inserted into the pRNAT H1.1 siRNA expression vector utilizing restriction enzyme sites at the end of the strands to express the siRNA. Stable cell line was constructed as follows: Bl6-BL6 cells were transfected with IDO2 shRNA or scrambled shRNA in a 12-well plate for 24 h and then reseeded to a 75 mm disk. Stably transfected IDO2 shRNA or scrambled shRNA B16-BL6 cells were selected and cloned by growth in a medium supplemented with 600 μg/mL of G418 (Sigma, St. Louis, MO).

### IDO2 mRNA quantification

Cells were lysed (Trizol reagent, Invitrogen) and total RNA was isolated according to the manufacturer's instructions. 1 μg of total RNA was synthesized to cDNA using reverse transcriptase (MMLV-RT, Invitrogen). The following primers sets were used for PCR amplifications: GAPDH, 5′-TGATGACATCAAGAAGGTGGTGAA-3′ (sense) and 5′-TCCTTG GAGGCCATGTAGGCCAT-3′ (antisense); IDO2, 5′-GTGGGGCTGGTCTATGAAGGTG-3′ (sense) and 5′-TGGTGGCAGCGGAGATAATGTA-3′ (antisense); IDO1, 5′-GGGCTTTGCTCTACCACATCCACT-3′ (sense) and 5′-ACATCGTCATCCCCTCGGTTCC-3′ (antisense). Real-time PCR reactions were performed in a Stratagene Mx3000P QPCR System (Agilent Technologies) using SYBR green PCR Master Mix (Life Technologies, Carlsbad, CA) according to manufacturer's protocol. Differences in gene expression were calculated using the ΔCt method.

### Western blot analysis

1.2×10^5^ cells were seeded into a 12-wheel plate and grown overnight. The cells were transfected with IDO2 siRNA or GL2 siRNA for 48 h. Cells were harvested, washed twice with ice-cold PBS, re-suspended in protein lysis buffer with complete protein inhibitor, and then the container was kept on ice for 30 min. Lysed cells were centrifuged at 15000 × RPM for 20 min at 4°C and the supernatant was collected and stored at −80°C for future use. Protein concentration was determined by Bio-Rad protein assay and 50 ug of each group cell lysate was separated on 12% SDS-PAGE, transferred to nitrocellulose membrane, blocked with 5% fat-free milk and 3% BSA in TBS-T (0.25% Tween 20), probed with a mouse anti-human IDO2 mAb that binds to both human and mouse IDO2 (Santa Cruz Biotechnology, Paso Robles, CA) and Monoclonal Anti-b-Actin Ab (Sigma, St. Louis, MO) according to the manufacturer's instructions, and visualized by an ECL assay (Pierce, Rockford, lL).

### NAD+ quantification

NAD+/NADH Quantification Kit (BioVision, Milpitas, CA; Catalog#K337-100) was used to measure NAD+ levels in the cells. Briefly, 1.2 ×10^5^ cells were seeded into a 12-wheel plate and grown overnight. The cells were transfected with IDO2 siRNA or GL2 siRNA for 48 h. The transfected cells were harvested and washed with ice-cold PBS, pelleted by centrifugation, and then NADH/NAD extraction buffer was used to lyse the cells by two freeze/thaw cycles. After the addition of NAD cycling buffer and NAD cycling enzyme mix to a total volume of 100 μl, NADt (total NAD including NADH and NAD) was detected in 50 μl of extracted samples. In order to degrade NAD+, the remainder sample was heated to 60°C for 30 min for NADH detection. The levels of NADH were measured in a similar fashion. NAD+ levels were calculated by subtracting NADH levels from NADt levels. Absorbance at 450 nm was measured on a microplate reader.

### Measurement of oxidative stress

Oxidative stress was determined by measuring the production of ROS using 5-(and-6)-carboxy-2′, 7′-dichlorodihydro-fluorescein diacetate (carboxy-H2DCFDA) (Sigma, St. Louis, MO) The nonpolar, nonionic H2-DCFDA is cell permeable and is hydrolyzed to nonfluorescent H2-DCF by intracellular esterases. H2-DCF is rapidly oxidized to highly fluorescent DCF when the peroxide presenting. Briefly, 1.2 ×10^5^ B16-BL6 cells were seeded in a 12-well plate and allowed to grow overnight. Cells were transfected with GL2 siRNA or IDO2 siRNA for 48 h and then harvested, pelleted by centrifugation, re-suspended with 5μl MH2DCFDA in DMSO for 30 min. Cells were detected using a BD FACSCalibur flow cytometer (BD Biosciences, Franklin Lakes, NJ) and analyzed by FlowJo software (Tree Star, Inc., Ashland, OR, USA).

### Cell proliferation assay

B16-BL6 cells were transfected with IDO2 siRNA or GL2 siRNA for 24 h in a 12-well plate, trypsinized, recounted and 5 ×10^2^ cells per well for each group were seeded in a 96-well plate in 200 μl complete medium. Cell proliferation was analyzed by MTT 96 h later. Briefly, 20 μl 3-(4,5-dimethylthiazol-2-yl)-2,5-diphenyltetrazolium (MTT) solution (5 mg/ml) was added into the culture medium of each well for 4 h of reaction in the incubator, then the medium was discarded. 100 μl DMSO was dissolved in the crystal by slowly shaking for 15 min. Absorbance at 490 nm was measured on a microplate reader.

### Cell apoptosis assay

Annexin V-FITC apoptosis detection kit (BD Pharmingen, San Diego, CA) was used to analyze apoptosis. 1.2 ×10^5^ B16-BL6 cells were seeded in a 12-well plate and allowed to grow overnight. Cells were transfected with GL2 or IDO2 siRNA for 48 h and then harvested, pelleted by centrifugation, re-suspended in binding buffer, and stained with annexin V-FITC and PI as recommended by the manufacturer. Cells were analyzed using a BD FACSCalibur flow cytometer and analyzed by FlowJo software. FITC/PI double-negative cells were identified as viable cells, FITC-positive cells were identified as being early apoptotic, FITC/PI double-positive cells were deemed late apoptotic, and PI-positive/FITC-negative cells were necrotic.

### Cell migration assay

Cell migration was measured by a scratch method. 1.2 ×10^5^ B16-BL6 cells were seeded in a 12-well plate and allowed to grow overnight. Cells were transfected with GL2 or IDO2 siRNA for 24 h. The surface of cells was scratched by a 10 ul tip, washed twice with PBS and allowed to migrate in DMEM without FBS. Images were taken at the beginning of scratch and 24 h later.

### Cell cycle analysis

B16-BL6 cells (1.2 ×10^5^) were cultured in a 12-well plate overnight, and then transfected with IDO2 siRNA or GL2 siRNA for 24 h. The cells were harvested, washed twice with PBS, pelleted by centrifugation, fixed in 75% ice-cold ethanol, and stored at −20°C overnight. The cells were washed twice with PBS and then re-suspended in 500 μl PI (20 μg/ml) (Sigma, St. Louis, MO) staining solution for 15 min at room temperature. The staining solution contained 0.1% Triton X-100 (Sigma, St. Louis, MO) and RNase A (Sigma, St. Louis, MO). Cells were detected using a BD FACSCalibur flow cytometer and analyzed by FlowJo software.

### siRNA expression vector treatment

C57BL/6 mice were treated with 50 μg of IDO2 or scrambled expression vectors in 1.0 ml PBS by hydrodynamic injection through the tail vein 3 days before cancer cell inoculation. For the inoculation, 2 ×10^5^ B16-BL6 cells were injected subcutaneously into the upper hind legs. At 7, 14 and 21 days after cancer cell inoculation, mice were treated again with 50 μg IDO2 or scrambled siRNA expression vectors in 1.0 ml PBS by hydrodynamic injection.

### Animal experiments

B16-BL6 cells were stably transfected with IDO2 shRNA or scrambled shRNA *in vitro*. The cells were harvested and washed twice in PBS, and viable, trypan blue-excluding cells were counted. Viable cells (2 ×10^5^) were re-suspended in a volume of 0.1 ml PBS then subcutaneously injected into the upper hind leg of each mouse. Tumor size was measured by caliper every other day when tumors appeared and the tumor volume was calculated using the following formula: tumor volume = 0.5 × (tumor width) × (tumor length). Tumor onset day was established as the point when tumor diameter reached 5 mm. All animal experiments were done in accordance with national standards, Canadian Council on Animal Care.

### Statistics

Data are presented as mean ± SD. Student's *t*-test (2-tailed) was used to determine differences between two means. For the comparison of multiple groups, one-way ANOVA test was applied. For all statistical analyses, differences with *p* values <0.05 were considered significant.

## SUPPLEMENTARY FIGURES


