# Supplementary material for: Gene silencing of indoleamine 2,3-dioxygenase 2 in melanoma cells induces apoptosis through the suppression of NAD+ and inhibits in vivo tumor growth
Source: Oncotarget. 2016 Apr 6;7(22):32329–40. doi: 10.18632/oncotarget.8617 (PMC5078016; doi:10.18632/oncotarget.8617)
Supplement: Supplementary file 1 [file oncotarget-07-32329-s001.pdf]

Gene silencing of indoleamine 2,3-dioxygenase 2 in melanoma cells induces apoptosis through the suppression of NAD+ and inhibits *in vivo* tumor growth

SUPPLEMENTARY FIGURES

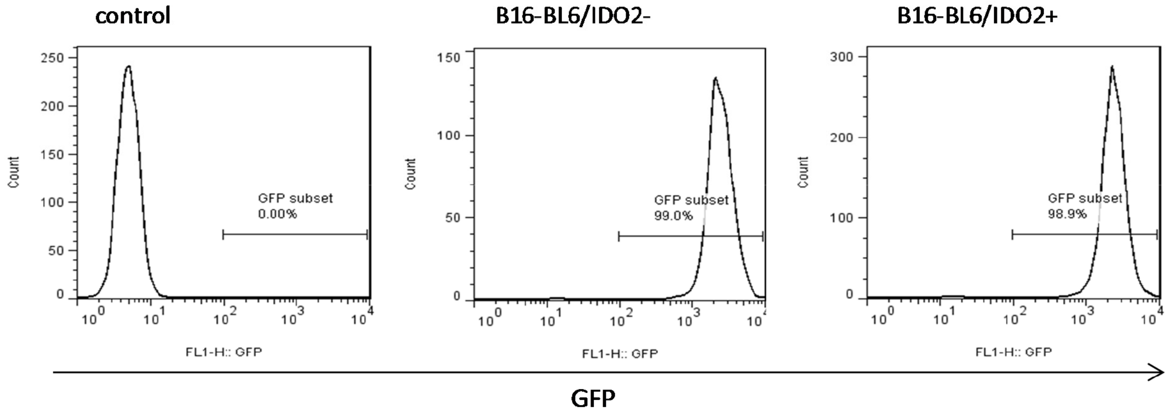

Supplementary Figure S1: Generation of IDO2 knockdown stable cell lines. B16-BL6 cells were transfected with scrambled shRNA or IDO2 shRNA that express GFP gene. The stable shRNA-expressing cells were then selected from the culture in the presence of G418 as described in Materials and Methods. The stable shRNA-expressing cells (GFP+ cells) were confirmed and sorted by flow cytometry.

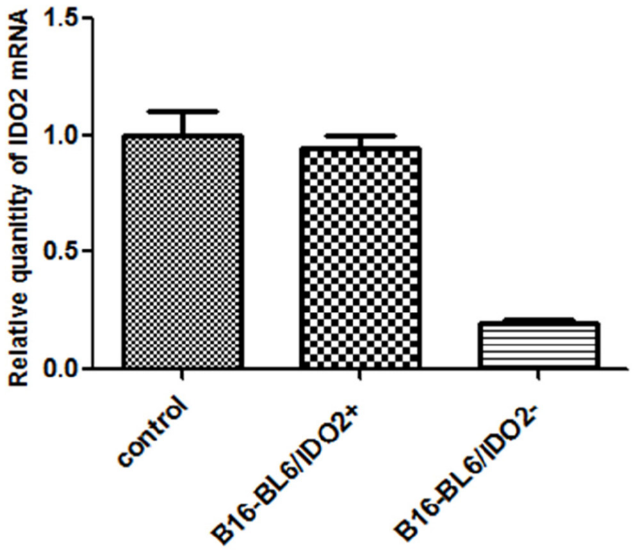

Supplementary Figure S2: Confirmation of IDO2 expression in IDO2 shRNA-transfected stable cells. Stable shRNA-expressing cells were generated as described above. IDO2 expression in IDO2 shRNA stable cells (IDO2-) and scrambled-shRNA transfected cells (IDO2+) were measured by qRT-PCR. Bars indicate the mean of three independent measurements ± SD (***p≤0.001).

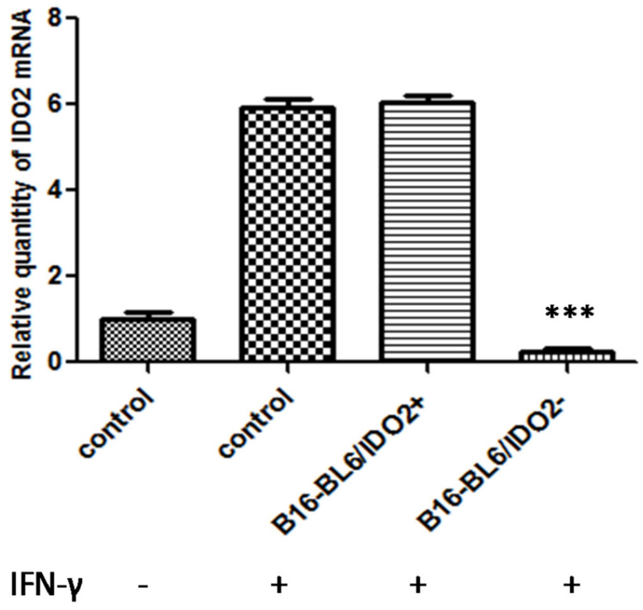

Supplementary Figure S3: IDO2 shRNA stable cells are resistant to stimulation by IFN-γ. IDO2 shRNA or scrambled shRNA expression stable cells were treated with IFN-γ (20 ng/m) for 24 h. IDO2 expression was detected by qRT-PCR. Bars indicate the mean of three independent measurements ± SD (***$p \leq 0.001$).
